# A Novel Approach to Facial Reanimation and Restoration Following Radical Parotidectomies †

**DOI:** 10.3390/jcm13082269

**Published:** 2024-04-14

**Authors:** Lucia Pannuto, Jun Yi Soh, Kwaku Duah-Asante, Shazrinizam Shaharan, Joseph Ward, Brian S. Bisase, Paul Norris, Isao Koshima, Charles Nduka, Ruben Yap Kannan

**Affiliations:** 1 Department of Plastic Surgery, Queen Victoria Hospital, East Grinstead RH19 3DZ, UK; sohjy86@hotmail.com (J.Y.S.); kwaku.duah-asante2@nhs.net (K.D.-A.); shaharas@gmail.com (S.S.); j.ward27@nhs.net (J.W.); cnduka@gmail.com (C.N.); ruben.kannan@nhs.net (R.Y.K.); 2 Department of Oral & Maxillofacial Surgery, Queen Victoria Hospital, East Grinstead RH19 3DZ, UK; brian.bisase@nhs.net (B.S.B.); paul.norris5@nhs.net (P.N.); 3 Department of Plastic Surgery, Hiroshima University Hospital, Hiroshima 734-0037, Japan; koushimaipla@gmail.com

**Keywords:** facial reanimation, radical parotidectomies

## Abstract

**Background**: Parotidectomies are indicated for a variety of reasons. Regardless of the indication for surgery, facial reanimation may be required because of facial nerve sacrifice or iatrogenic damage. In these cases, facial restoration performed concurrently with ablative surgery is considered the gold standard, and delayed reanimation is usually not attempted. **Methods:** A retrospective review of all patients who underwent parotidectomies from 2009 to 2022 in a single institution was performed. Indications, surgical techniques, and outcomes of an algorithmic template were applied to these cases using the Sunnybrook, Terzis scores, and Smile Index. A comparison was made between immediate vs. late repairs. **Results:** Of a total of 90 patients who underwent parotidectomy, 17 (15.3%) had a radical parotidectomy, and 73 (84.7%) had a total or superficial parotidectomy. Among those who underwent complete removal of the gland and nerve sacrifice, eight patients (47.1%) had facial restoration. There were four patients each in the immediate (*n* = 4) and late repair (n = 4) groups. Surgical techniques ranged from cable grafts to vascularized cross facial nerve grafts (sural communicating nerve flap as per the Koshima procedure) and vascularized nerve flaps (chimeric vastus lateralis and anterolateral thigh flaps, and superficial circumflex perforator flap with lateral femoral cutaneous nerve). **Conclusions:** The algorithm between one technique and another should take into consideration age, comorbidities, soft tissue defects, presence of facial nerve branches for reinnervation, and donor site morbidity. While immediate facial nerve repair is ideal, there is still benefit in performing a delayed repair in this algorithm.

## 1. Introduction

A parotidectomy is the partial or complete removal of the parotid gland, the largest salivary gland in the body. In a radical parotidectomy, both the parotid and the facial nerve are excised. This is often indicated in cases of advanced malignancy where the facial nerve has been invaded by a tumor, or if there is preoperative impairment of facial nerve function. In total parotidectomies, the entire gland is excised while the facial nerve is preserved [1]. Mucoepidermoid and adenoid cystic carcinomas are the most prevalent malignancies of the parotid gland; however, up to 80% of parotid neoplasms are benign in nature, with the most common being pleomorphic adenomas and Warthin’s tumors [2]. Other indications for parotidectomy include infections, such as tuberculosis and toxoplasmosis, caseating granulomas, and congenital malformation [3].

Facial nerve sacrifice invariably leaves the patient with varying degrees of motor dysfunction and paralysis. The surgery often inflicts volume loss across the angle of the mandible, having huge aesthetic ramifications. Facial nerve function is essential for eye closure, tear production, facial emotional expression, and speech. Therefore, injury impacts these functions and has massive psychosocial implications for the patient [4]. The goal of facial reanimation is to restore facial functions, facial symmetry, social interaction, and quality of life [5]. The current literature supports nerve reconstruction being performed as soon as possible [6,7,8,9,10,11].

This is said to be due to a decrease in the number of motor units as early as two months post-denervation, as well as atrophy of the target units [12,13]. However, there may be some advantages to delaying the surgical procedure, such as oncological safety, pending response to radiotherapy, length of surgery, patient fitness, and the involvement of the multidisciplinary team to carefully plan the surgical teams. In this study, we explore the functional results of early and delayed facial reanimation following parotidectomy after the application of our algorithm.

## 2. Materials and Methods

The primary aim of this review of clinical practice was to develop an algorithm to guide intra-operative decision making when performing a radical parotidectomy, with the intention of restoring both form and function; the secondary outcome was to compare immediate and delayed reconstruction, up to eighteen months post-parotidectomy.

Medical records of patients who underwent facial reanimation after parotidectomy at our institution between 2009 and 2022 were reviewed. All patients granted consent for the operative treatment and photographs in accordance with the Declaration of Helsinki. These cases were treated in collaboration with the Maxillo-facial team, and all reconstructions were performed by the senior authors (B.S., P.N, C.N.D. and R.Y.K).

A retrospective cohort study was performed, and data regarding demographic details, type of surgery, etiology, timing of reconstruction, duration of follow-up, ancillary procedures, complications, and outcomes were assessed. Data were extracted from the hospital database.

Inclusion criteria were all patients who underwent parotidectomy and concurrent facial reanimation at Queen Victoria Hospital between 1 January 2009, and 1 January 2022. Exclusion criteria were patients with incomplete data or follow-up shorter than one year after facial reanimation.

Tumor surveillance was conducted by our multidisciplinary team, including maxillo-facial surgeons, oncologists, and radiotherapists. Follow-up for facial reanimation was conducted by our facial palsy team, including plastic surgeons, facial therapists, and specialized nurses.

Post-operative photographs and videos were taken by our photographic team at regular follow-ups. Results were evaluated by two surgeons independently (L.P. and J.Y.S.) based on the Sunnybrook score, Terzis score, and Ackerman Smile Index. The Sunnybrook score was calculated as “Voluntary movement score—Resting symmetry score—Synkinesis score”, from 0 to 100 [14]. The Terzis score was used for grading the smile and the overall aesthetic outcome, from 0 to 5 [15], and the Smile Index as a measure of the “intercommissural width/interlabial gap height” during smile [16]. In all cases of radical parotidectomy and immediate reconstruction, the initial score was assessed on pictures and videos taken right after the oncological resection at the first outpatient follow-up; thus, the improvement in facial nerve function was attributed to the repair performed. In all cases of delayed reanimation, pre-operative scores were registered based on the last follow-up before the reconstructive procedure. Post-operative results were assessed at the last post-procedural follow-up visit.

**Figure 1:**
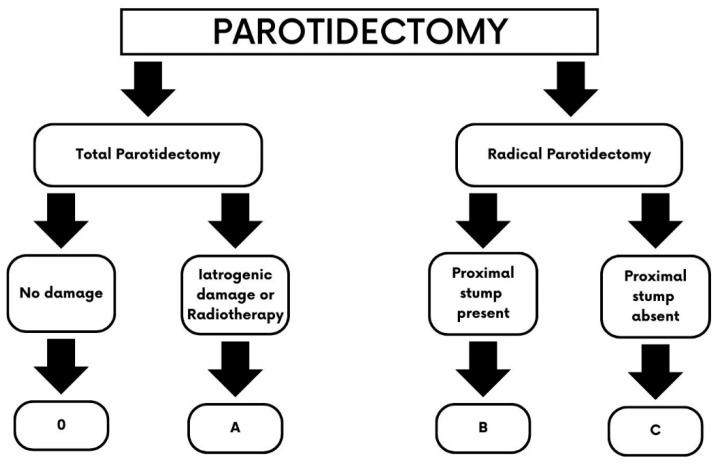
**East Grinstead classification** for facial restoration after parotidectomy. All patients are divided into two groups based on whether they undergo a total or radical parotidectomy. In case of a total parotidectomy and preservation of the facial nerve, patients are classified as type 0. However, if the nerve is accidentally damaged during the procedure or adjuvant radiotherapy is planned, they are categorised as type A. When a radical parotidectomy is performed and the facial nerve is sacrificed, patients are classified as type B if the proximal stump of the nerve is preserved, or type C in case the nerve is resected at the stylomastoid foramen, and no proximal stump is available for reanimation.

All patients who underwent parotidectomy and sacrifice of the facial nerve or iatrogenic damage were divided in 3 groups according to our algorithm (Figure 1). We defined immediate facial reanimation as being performed at the same time as parotidectomy or within 1 week. Delayed repairs were defined as being performed after 1 week to 18 months from oncological resection.

## 3. East Grinstead Classification

The cohort was further sub-divided into those having total and radical parotidectomy.

The total parotidectomy patients are further categorised into those having no nerve damage and those with iatrogenic nerve damage or who are undergoing adjuvant radiotherapy. The radical parotidectomy patients are further subdivided into either having a proximal stump present or not (Figure 1).

**Figure 2:**
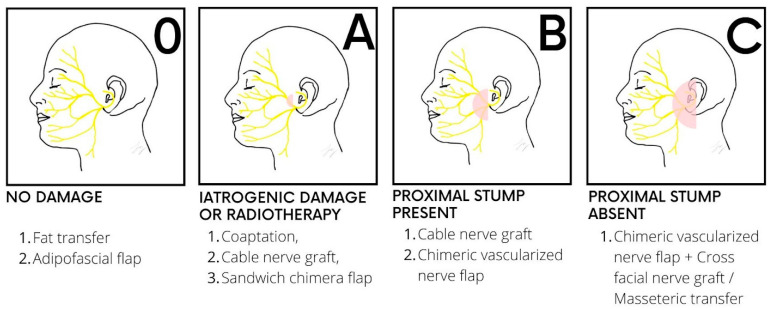
Reconstructive algorithm for facial restoration after parotidectomy, based on the East Grinstead classification. Type (0): Patients who benefit from lipofilling or an adipo-fascial flap for volume restoration. No facial reanimation is required. Type (A): The facial nerve can be repaired through coaptation or using an interposition nerve graft such as the great auricular nerve or the sural nerve; in cases where radiotherapy is planned, a free flap can be placed around the nerve (“sandwich” flap) to prevent tissue atrophy. Type (B): A chimeric vascularized nerve flap is usually required, such a vastus lateralis and anterolateral flap with the motor nerve of the vastus lateralis that acts as a cable graft between the proximal stump and the distal branches of the facial nerve. When the volume deficit is not a concern, a cable graft may be sufficient. Type (C): When no proximal stump is available, a cross-facial nerve graft and/or a masseteric transfer is required together with a vascularized nerve flap.

The classification for facial reanimation after parotidectomy is based on the East Grinstead Classification, which comprises four groups:

- Type 0: patients who underwent total parotidectomy with no nerve damage.
- Type A: patients who underwent total parotidectomy with iatrogenic damage to the facial nerve.
- Type B: patients who underwent radical parotidectomy with sacrifice of the facial nerve and residual proximal stump of the nerve available for repair.
- Type C: patients who underwent radical parotidectomy with sacrifice of the facial nerve up to the nerve canal to reach a tumour-free proximal nerve stump.
- In each case, neurology, volume, support, and coverage are addressed [17] (Figure 2).

**Figure 3:**
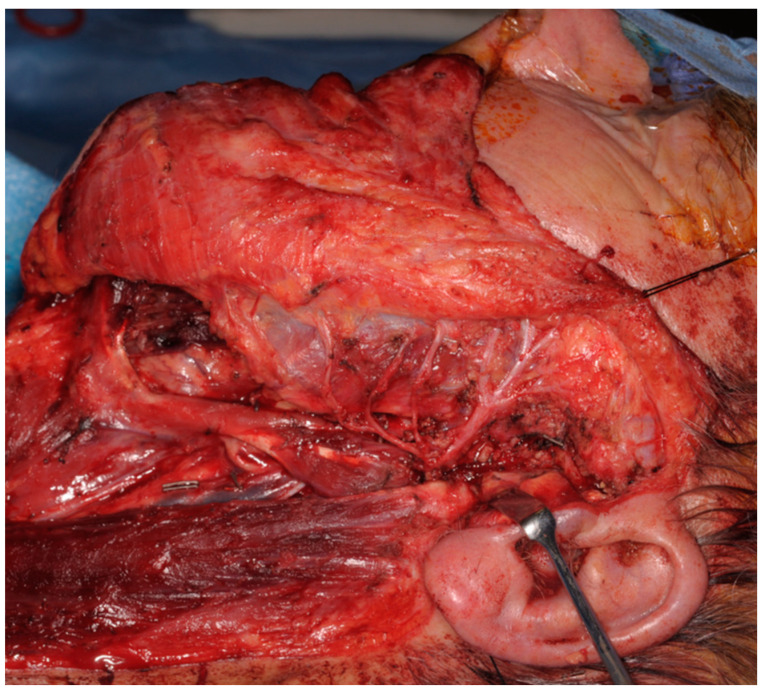
**Intra-operative picture of type 0 defect.** Total parotidectomy with preservation of the facial nerve. In case of iatrogenic damage or adjuvant radiotherapy, patients are classified as type A.

For type 0, the contour defect is usually mild and, in most instances, is managed conservatively, but if requested, second-stage lipofilling with the use of a facial nerve monitor can be offered. As neurology is not affected, no further intervention is required. A free flap can also be considered in cases where more volume is needed, but this is a discussion that is made pre-operatively, in cases of total parotidectomy with facial nerve preservation (Figure 3). In cases of type A defects, the contour defect may be similar to that of type 0 patients, but while fat transfer is the simpler option in terms of volume restoration, it is to be considered in a second stage once direct facial nerve coaptation or cable grafts to repair iatrogenic facial nerve injuries are performed as the first procedure. Again, there remains the risk of damaging the facial nerve during the second stage of lipofilling, and care must be taken with the use of a facial nerve monitor during this process.

An alternative option is a free chimera flap, such as a “sandwich” anterolateral thigh—vastus lateralis flap. This is advantageous as it reduces the risk of radiation damage to the peri-neural soft tissue atrophy and the facial nerve, should adjuvant radiotherapy be required. This is our preferred option as it provides both a vascularised nerve for reanimation and structural fat in a single setting, which, in the long term, minimises subsequent risk to the facial nerve.

**Figure 4:**
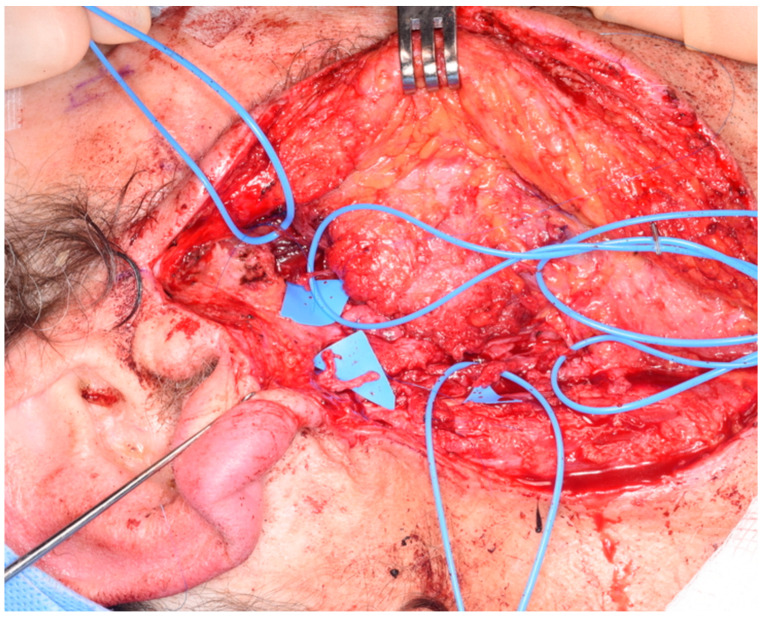
Intra-operative picture of a type B defect. The parotid gland has been completely removed together with the main branches of the facial nerve. The proximal stump has been preserved.

For type B defects, the volume deficit is usually greater, and a free flap is our standard of choice (Figure 4). This is harvested with the accompanying nerve that is used as a vascularized interposition graft. An example is the anterolateral thigh flap, which is raised as a chimera with the vastus lateralis muscle and its motor nerve branch, as a vascularized nerve graft. The vascularized nerve graft is cable grafted from the proximal stump of the facial nerve to the distal branches. More support can be added with fascia slings to the oral commissure or ancillary procedures. In some instances, in type B defects, if the volume deficit is minimal and the patient is not concerned with this prospective contour defect, a simpler cable graft from the sural or greater auricular nerve is used instead to restore facial nerve continuity.

**Figure 5:**
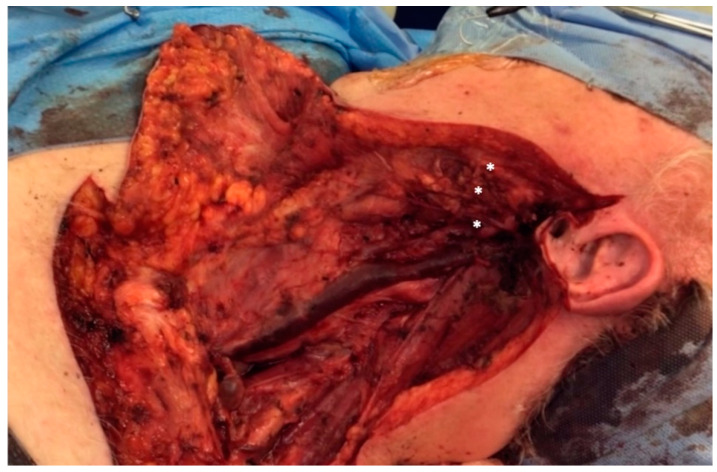
**Intra-operative picture of type C defect.** Radical parotidectomy with complete sacrifice of the facial nerve and no proximal stump available for coaptation. The distal branches of the cervical, marginal mandibular and bucco-zygomatic branches are marked with asterisks (*).

However, when the proximal stump of the facial nerve is not available for interposition nerve grafting, different available donor nerves need to be considered as sources of reinnervation. For this reason, type C patients need a masseteric transfer and/or a cross facial nerve graft in addition to a free flap or fat transfer for volume restoration (Figure 5). Again, this can be considered in one or two stages, as per an informed decision with the patient. In case of a two-stage procedure, either a nerve-to-masseter (NTM) transfer or a cross-facial nerve graft (CFNG) is performed first, with the consideration of secondary lipofilling and the use of a facial nerve monitor. However, our preference is to use a vascularized nerve flap [18] instead, which again provides the benefits of a vascularized nerve plus structural fat, which allows the overlying facial skin to be secured to it for optimal volume contouring.

## 4. Statistical Analysis

Initial and post-operative Sunnybrook score, Terzis score, and Smile Index were recorded for each patient in the immediate repair group and in the delayed repair group. Intra-rater reliability was assessed using intraclass correlation coefficients (ICCs) [19]. Quantitative variables were analysed using the two-tailed student’s *t* test for independent samples. Statistical analysis was performed using the IBM SPSS 25.0 software (IBM Corp, Armonk, NY, USA); *p* < 0.05 was considered statistically significant.

## 5. Results

From 2009 to 2022, a total of ninety parotidectomies (n = 90) were performed at our institution. Among these, 73 (84.7%) were total or superficial parotidectomies, and 17 (15.3%) were radical parotidectomies. Eight patients (47.1%) of those in whom the facial nerve was sacrificed underwent facial reanimation and were added to the study. Half of these, four (50%), had an immediate repair, with facial restoration at the same time as the oncological resection. The other half, four (50%), had a delayed repair (mean 9.75 ± 5.5 months post-onset). All patients were followed up for at least one year since facial reanimation, except for one patient in the immediate group who died due to tumour progression and was therefore excluded from the study. The mean follow-up length in the immediate repair group was 40 ± 24.98 months, while in the delayed repair group it was 23 ± 16.85 months. Demographic characteristics of the two groups and the results are illustrated in Table 1.

**Figure 6:**
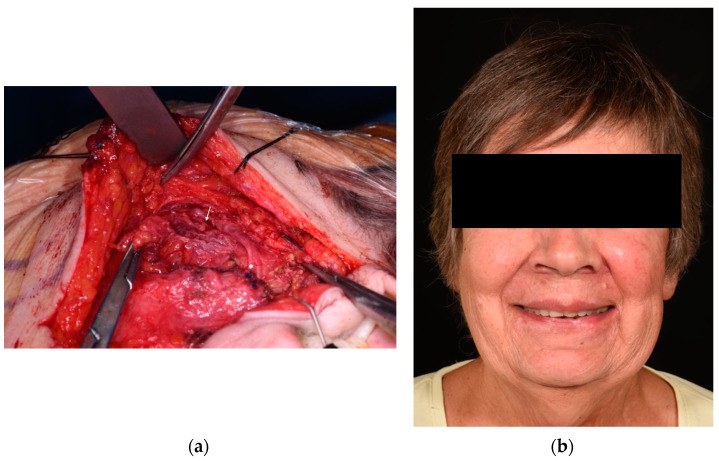
Patient who underwent radical parotidectomy and immediate facial reanimation (type B defect). Intra-operative picture of a 71-year-old woman who underwent a left radical parotidectomy for an adenocarcinoma with sacrifice of the bucco-zygomatic branch (**a**). In the intra-operative picture after resection of the tumour, the distal branch of the nerve is marked with an arrow and the proximal stump with an asterisk. Immediate repair was performed with a great auricular nerve cable graft and fascia lata sling. The volume deficit was mild and not treated. One-year post-operative pictures show a Sunnybrook score of 100, Terzis score of 4, and Smile Index of 10.5 (**b**).

In the immediate group, two patients had type B defects and one, type C. In one of those with a type B defect, a great auricular nerve cable graft was used to restore motor function to the left hemi-face (Figure 6), while in the others, a chimeric vastus lateralis and anterolateral flaps with the motor nerve used as interposition grafts were performed. In the type C case, the masseteric nerve was used as the donor motor source, with the motor branch of the vastus lateralis functioning as the neural component of the flap chimera.

**Figure 7:**
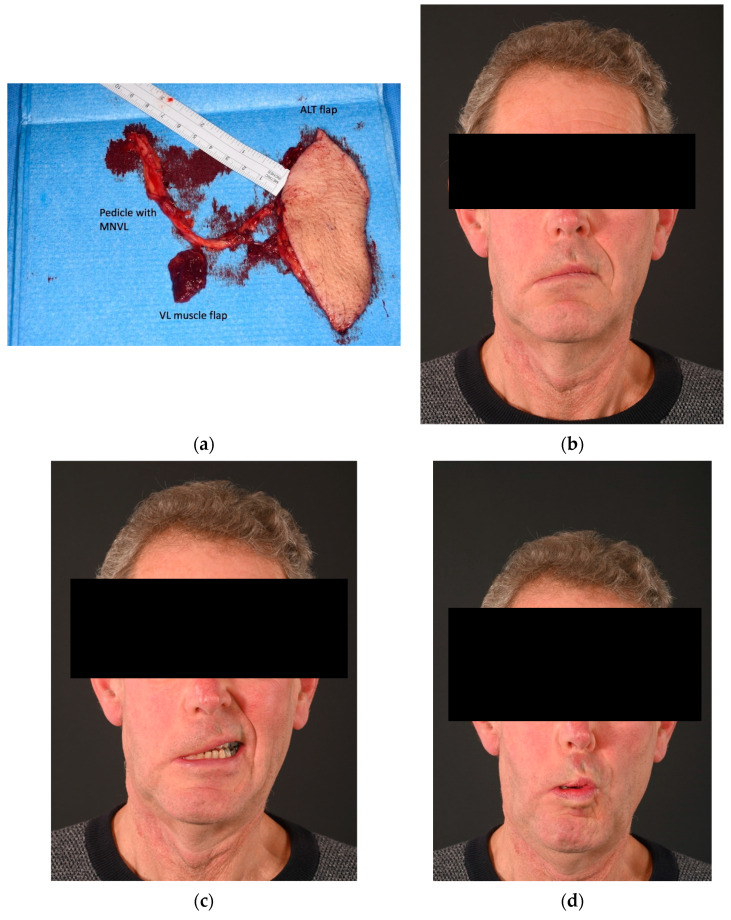
Patient who underwent radical parotidectomy and delayed facial reanimation (type B). Sixty-three-year-old patient who underwent a right radical parotidectomy for an acinic cell carcinoma and selective neck dissection. Reconstruction was performed after 5 months with a chimeric vastus lateralis and anterolateral flap (**a**). One-year post-operative pictures show a Sunnybrook score of 33%, Terzis score of 2, and Smile Index of 9 (**b**–**d**). The clinical improvement in this case was mainly to the lower trunk of the facial nerve: buccinator, lower lip depressor and platysmal function.. However, no significant return of smile has been exhibited at one-year post-op. This patient may require a lengthening temporalis myoplasty procedure in the future, for smile reanimation. MNVL, motor nerve to vastus lateralis; ALT, anterolateral thigh; VL, vastus lateralis.

**Figure 8:**
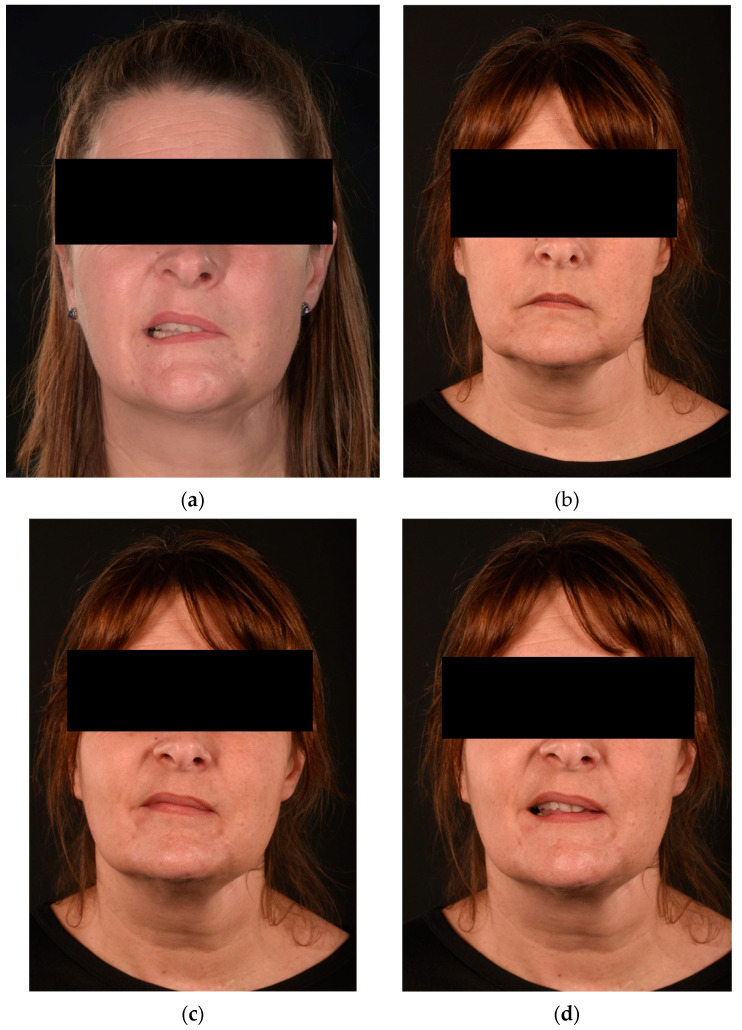
Patient who underwent radical parotidectomy and delayed facial reanimation (type C). Forty-six-year-old patient treated in another country for a complicated parotid cyst infection and subsequent left parotidectomy with cauterization of the facial nerve at the exit of the stylomastoid foramen (**a**). Facial reanimation has been performed after 17 months with an intra-oral vascularized cross facial nerve flap (sural communicating nerve flap) between the levator labii superioris branches of each side. A masseteric nerve transfer to the buccal and zygomatic branches of the left side has also been performed. Post-operative pictures at 14 months show an optimal facial repose and improved smile symmetry, contributing to an overall Sunnybrook score of 70%, Terzis score of 3, and Smile Index of 7.5 (**b**–**d**).

In the delayed group, one patient had a type B defect, and a chimeric vastus lateralis and anterolateral flap with the motor nerve to the vastus lateralis was the reconstructive choice (Figure 7), while the other three patients had type C defects. For these, the senior author performed a chimeric superficial circumflex perforator (SCIP) flap with lateral cutaneous femoral nerve coaptation to the masseteric nerve, and two cases of an intra-oral vascularized cross facial nerve flaps: the “Koshima” procedure [20], wherein the sural communicating nerve (SCoNe) with its accompanying artery, the superficial lateral sural artery, was harvested and anastomosed to the superior labial vessels [21] (Figure 8; Supplementary Materials Videos S1 and S2).

The mean post-op Sunnybrook score was 48.75 for the delayed group and 53.67 for the immediate group. The mean post-op Terzis score in the delayed group was 2.75 and in the immediate group was 2.65. As per the Smile Index, this was 8.75 and 5.50 in the delayed group and in the immediate group, respectively. No statistically significant differences were found between the two groups (Table 2), but it is to be noted that this is a pilot study, and more cases are required to derive a more concrete result.

**Figure 9:**
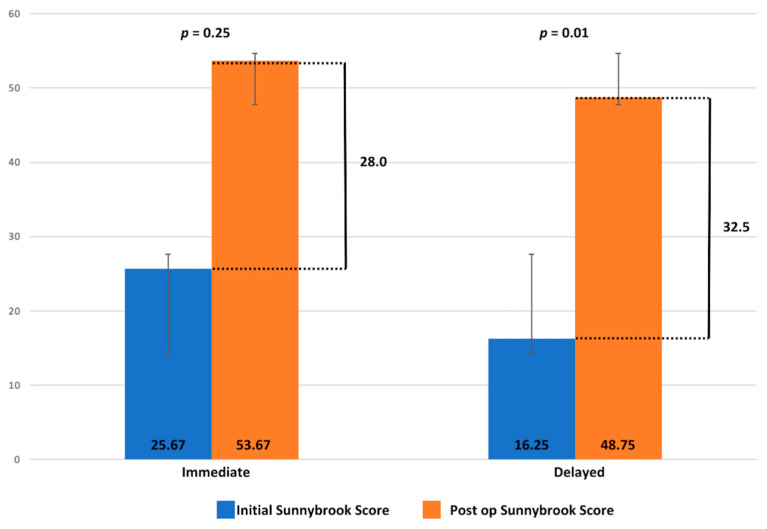
Sunnybrook score improvement of immediate and delayed reanimation after radical parotidectomy. A statistically significant difference is shown between the initial and post-operative Sunnybrook scores in the delayed repair group with *p* < 0.05.

In terms of quantitative improvement of the Sunnybrook score, this was 32.5 ± 12.79 for the delayed group and 28 ± 22.91 for the immediate group. Comparing the initial and post-op Sunnybrook scores of the patients in the immediate group, there was no significant improvement (*p* = 0.25), whereas for the patients in the delayed group, this was significant (*p* < 0.05) (Figure 9). However, it should be noted that all pre-op patients in the acute group had normal or near-normal facial function, compared to those who presented later. This represents a limitation of this study. In terms of inter-rater reliability, the ICCs were 0.977, demonstrating excellent inter-rater reliability (*p* < 0.001).

Ancillary procedures were performed to gain symmetry in four cases. These consisted mainly of oculoplastic procedures, lipofilling, fascia latae sling, and botulin toxin injection for the treatment of synkinesis and contralateral hyperkinesis. As per post-operative complications, one patient was taken back to the theatre for haematoma evacuation and one for drainage of a localized abscess, both in the delayed group. One patient in the immediate group had multiple comorbidities, including diabetes mellitus type II, and died after four years from other medical reasons.

## 6. Discussion

Immediate facial reanimation is commonly considered the gold standard to try to achieve the best function possible after parotidectomy [22,23]. However, when facial nerve sacrifice is required, ablative surgery can already be a challenge for both patient and surgeon due to severe malignancy or advanced disease. It is reported that only 25.5% of patients undergo a concurrent reinnervation procedure and 24.0% undergo a concurrent reanimation procedure after total parotidectomy with sacrifice of the facial nerve [24]. Based on this, the goal of our study was to assess if there is still benefit in attempting delayed facial reanimation and restoration after radical parotidectomies. The outcomes of the delayed cases were compared to immediate reconstructions against the Sunnybrook scores, Terzis scores, and Smile index scores based on our current algorithm. We defined an immediate reconstruction as performed within a week and delayed reconstruction as those performed between a week and eighteen months post-ablative surgery, with eighteen months being the cut-off for reinnervation and muscle preservation.

While the advantages of immediate facial reanimation are well described in medical literature, in real terms, intraoperative assessment of distal facial nerve branches is difficult in delayed cases due to the Wallerian degeneration and scarring of previous surgery. Denervation also has a detrimental effect on the distal nerve stump and the muscle targets as an independent factor deteriorating functional outcomes after nerve repair [25,26]. However, other factors can have an even greater impact on such complex cases, such as advanced age and poor long-term prognosis.

While our preference is to perform single-stage facial reanimation and volume restoration, a two-stage approach with a delayed reconstruction may be beneficial in selected cases wherein the histological margins are in question, those with significant co-morbidities that preclude prolonged operating times, or in referred patients who were not offered facial reanimation during ablative surgery by our surgical oncology counterparts. Therefore, it is important to adopt a flexible mindset in this regard.

Gur et al. illustrated a general approach for the management of facial paralysis based on static or dynamic correction of asymmetry and duration of the paralysis [27]. However, facial restoration after parotidectomies requires a specific subset of surgical techniques that allow the surgeon to correct neurology, coverage, volume, and support at the same time when needed [28]. Ciolek et al., on the other hand, present their experience utilizing the anterolateral thigh (ALT) free flap, orthodromic temporalis tendon transfer (OTTT), and facial nerve cable grafting to re-establish form and function after radical parotidectomies [29]. Similarly, Elliott et al. centered their reconstructive algorithm on the composite/chimeric anterolateral flap, providing both a vascularized nerve as well as fascia lata for both static and dynamic reanimation [30], as did Hasmat et al. using the chimeric vastus lateralis and anterolateral flap harvested with the motor nerve [31].

In our case series, instead of focussing on a single flap type, we utilised multiple different vascularized nerve flaps or grafts based on the availability of the residual facial nerve and the patient’s body phenotype. Other factors include the volume of soft tissue needed, patient comorbidities, prognosis, donor site characteristics, and multidisciplinary team input. In some instances, the volume defect is not a concern for the patient, and even for those with types B and C defects, restoration of neural continuity is emphasized. Ancillary procedures can then be considered later. It is to be noted in this study that facial reanimation does not only pertain to smile restoration but also functional outcomes, such as eye closure, lip pucker, and buccinator function as well.

While we attempt to compare outcomes between early and delayed presentations after radical parotidectomies, the numbers in this pilot study are too small to reach any conclusion, but this was performed nevertheless, to illustrate that facial restoration, even in the delayed group, is a worthwhile endeavour, although smile restoration may not be optimal in all cases. One patient was excluded from this study, a patient who died three months after radical parotidectomy and immediate reconstruction due to tumour progression (SCC of the ear). This case did not fit into our inclusion criteria of having at least one-year follow-up.

While we prefer the use of free tissue transfers, other options, such as a cervicofacial rotation advancement flap [5] or dermal substitutes [32] must be considered with delayed facial reanimation (within 18 months post-onset). Overall, this study poses more questions than answers due to the small sample size of patients who have had facial nerve sacrifice. Moving forwards, we invite a multicentre prospective trial with larger study samples and outcomes to consolidate these findings.

## 7. Conclusions

In conclusion, we propose a new classification for facial reanimation after parotidectomy based on the extent of the facial nerve sacrifice or damage. Using this template, a practical reconstructive algorithm has been put forward. It is also to be noted that where possible, immediate facial restoration is the gold standard; however, a delayed approach still allows for good functional outcomes and can be considered in selected cases.

## Figures and Tables

**Table 1:** Patient demographics and results.

Group	Age	Gender	Side	Aetiology	Defect Type	Months to Reanimation	Procedure	Adjuvant Radiotherapy	Months of Follow-Up	Ancillary Procedures	Initial SB Score	Post-op SB Score	SB Overall Improvement	Post-Op Terzis Score	Post-Op Smile Index
Immediate															
1	71	F	Left	Adenocarcinoma	B	0	GAN cable graft	No	12	Fascia lata sling	77%	100%	23%	4	10.5
2	46	F	Right	Adenocarcinoma	B	0	Chimeric ALT-VL flap + MNVL	Yes	60	Botulin injections	0%	53%	53%	3	6
3	54	F	Right	Infection	C	0	Chimeric ALT-VL flap + MNVL to V3m	No	48	No	0%	8%	8%	1	0
Delayed															
1	46	F	Left	Infection	C	17	Vascularized CFNG (SCoNe flap) + MT	No	14	No	39%	70%	31%	3	7.5
2	61	F	Right	Adenoid cystic carcinoma	C	11	Vascularized CFNG (SCoNe flap)	No	18	Fascia lata sling, Lipofilling	4%	47%	43%	3	7.5
3	41	M	Right	Preauricular SCC	C	6	Chimeric SCIP flap + LCFN to V3m	Yes	48	Oculoplastic procedures	4%	45%	41%	3	11
4	63	M	Right	Acinic cell carcinoma	B	5	Chimeric ALT-VL flap + MNVL	No	12	No	18%	33%	15%	2	9

SB: Sunnybrook; GAN: great auricular nerve; ALT-VL: anterolateral-vastus lateralis flap; MNVL: motor nerve to the vastus lateralis; V3m: masseteric branch of the mandibular nerve; CFNG: cross-facial nerve graft; SCoNe flap: sural communicating nerve flap; MT: masseteric nerve transfer; SCIP: superficial circumflex perforator flap; LCFN: lateral cutaneous femoral nerve.

**Table 2:** Post-operative results of patients who underwent radical parotidectomy and immediate or delayed facial reanimation.

	Post-op Sunnybrook Score (Mean ± SD)	Post-op Terzis Score (Mean ± SD)	Post-op Smile Index (Mean ± SD)
Group			
Immediate	53.67 ± 46	2.65 ± 1.53	5.5 ± 5.27
Delayed	48.75 ± 15.46	2.75 ± 0.5	8.75 ± 1.66
*p* ^a^	0.42	0.46	0.14

Results are written as mean ± SD. ^a^: calculated with Student’s *t* test.

## Data Availability

As the data from this review of clinical practice was registered as a clinical audit under the Clinical Audit Department at the Queen Victoria Hospital, East Grinstead, any data is available only on request to the Queen Victoria Hospital, due to restrictions of clinical governance and data protection.

## Supplementary Materials

The following supporting information can be downloaded at: https://www.mdpi.com/article/10.3390/jcm13082269/s1, Video S1: Pre-operative video of patient who underwent radical parotidectomy and delayed reanimation (type C defect). A 61-year-old woman at the first follow-up after radical parotidectomy for a right adenoid cystic carcinoma with sacrifice of the facial nerve, temporalis muscle, masseter muscle and radical neck dissection. Her initial Sunnybrook score was 4; Video S2: Post-operative video of patient who underwent radical parotidectomy and delayed reanimation (type C defect). The same patient of Video S1 underwent delayed reanimation 11 months after her radical parotidectomy. The post-operative video at 18 months follow-up after a vascularized cross-facial nerve graft using the sural communicating nerve (SCoNe) along with a fascia lata sling shows a Sunnybrook score of 47, Terzis score of 3, and Smile Index of 7.5.
